# A Systematic Review on the Applications of Uppaal

**DOI:** 10.3390/s25113484

**Published:** 2025-05-31

**Authors:** Iwona Grobelna, Krystian Gajewski, Andrei Karatkevich

**Affiliations:** 1 Institute of Automatic Control, Electronics and Electrical Engineering, University of Zielona Góra, 65-516 Zielona Góra, Poland; 2 Department of Applied Computer Science, AGH University of Science and Technology, 30-059 Kraków, Poland

**Keywords:** control systems, formal verification, model checking, modeling and verification, Uppaal

## Abstract

This paper presents a systematic review on possible applications of the Uppaal tool. This tool, an integrated environment for the modeling, validation, and verification of real-time systems modeled as networks of timed automata, is currently used in various domains of science and engineering. A systematic review of the literature from the years 2022 and 2023 was conducted following the Preferred Reporting Items for Systematic Reviews and Meta-Analyses (PRISMA) procedure. The aim was to identify the current application areas of various versions of the Uppaal tool, including CORA, TIGA, SMC, and Stratego. A total of 188 studies were included in the review. Quantitative information on the distribution of research papers regarding access options, scientific databases, types of papers, and geographical location was obtained. This review highlights the need for further development of the Uppaal tool. In addition, it includes a brief comparison with other mainstream formal validation tools, explores the applicability of different Uppaal versions, and offers practical guidelines for version selection. Finally, key open challenges and their potential solutions are discussed to support future research and tool enhancement.

## 1. Introduction

Formal verification has been gaining popularity for decades [1], both in the scientific world and in industry. Mathematical models are analyzed and verified, gaining the most benefit in the early stages of system development. One of the most efficient methods is model checking [2]—an automatic technique for the verification of reactive systems against user-defined requirements, usually expressed as temporal logic formulas. In symbolic model checking, it can be guaranteed can that the system considered satisfies the specified requirements [3]. Otherwise, appropriate counterexamples are generated that include traces leading to undesired situations, simplifying error finding. In turn, statistical model checking [4] combines simulation and statistical methods to gain statistically valid results. It enables the prediction of system behavior with high confidence. There are numerous model checking tools available, including NuSMV [5] and its successor nuXmv [6], SPIN [7], PRISM [8], and Uppaal [9], but also some less popular ones like HyTech [10], Ymer [11], and Zing [12]. There are also very diverse application areas that benefit from using available model checkers, from power systems [13] to Industry 5.0 with Digital Twins [14] or IoT [15] and fusion with artificial intelligence [16]. In this review article, we focus on the Uppaal tool and examine its impact on recent and ongoing research. Formal modeling and verification are popular emerging topics. A very recently published survey [17] identified four key research questions focusing on tool characteristics, modeling methods, verification techniques, and application domains. Our review complements that work by providing a wider perspective, expanding the scope by including a larger set of publications and obtaining some new interesting results on the application of the Uppaal tool.

The Uppaal tool [9] is a widely known environment for the verification, modeling, and validation of real-time systems. It is maintained and developed by two scientific institutions: Uppsaala University, Sweden, and Aalborg University, Denmark. Due to its wide range of capabilities, Uppaal has dedicated branches for different usage. For example, Uppaal SMC [18] is used for statistical model checking, Uppaal Stratego [19] is used to analyze strategies, and Uppaal TIGA [20] is used to solve games. The application areas of Uppaal are as diverse as its versions.

The purpose of this study was to investigate the current application of the Uppaal tool in various fields of science and to find some general statistics on its usage. A systematic review was performed following the PRISMA procedure. The following widely known scientific databases were searched: IEEE Xplore, Elsevier, Springer, ACM, MDPI, and Google Scholar. Of the 1040 primary papers found, we identified 188 suitable works following the PRISMA procedure [21] and the specified inclusion/exclusion criteria. Five research questions were defined to obtain qualitative and quantitative results. By responding to these questions, our aim was to provide summaries and insights into possible further developments of the Uppaal tool.

The contributions of this paper are as follows:

1. Presenting a systematic review of recent research works using Uppaal;
2. Obtaining some quantitative information on the distribution of research papers regarding access options, scientific databases, types of papers, and geographical location;
3. Analyzing the applicability and capabilities of different Uppaal versions supported by demonstrative case studies;
4. Proposing practical guidelines for selecting the appropriate Uppaal version based on the application context;
5. Identifying the current challenges and outlining potential future research directions and tool enhancements.

The remainder of the paper is structured as follows. Section 2 provides some background information on the Uppaal tool, especially focusing on the different versions. Section 3 describes the research methodology and defines the main research questions. Section 4 presents the obtained results, considering both the specified research questions and some statistics. Section 5 offers an extended discussion, including a brief comparison with other mainstream formal validation tools, an analysis of the applicability of various Uppaal versions, practical guidelines for version selection, and the identification of open challenges and their possible solutions. Finally, Section 6 concludes the article, indicates future perspectives, and lists the limitations of this study.

## 2. Background

Let us briefly summarize the modeling and verification tool Uppaal and its versions. Uppaal, first released in 1995, is an “integrated tool environment for modeling, validation, and verification of real-time systems modeled as networks of timed automata, extended with data types” (according to the home page of Uppaal, https://uppaal.org/, last accessed 12 February 2025). It has been shaped by the need of industry for model-based validation, performance evaluation, and synthesis [22]. It is free for academic use; any other use requires a license. The Uppaal models are specified in the form of hybrid timed automata, connected in networks, and extended with clock and data variables. The main functions, i.e., simulation and model checking, facilitate checking model behavior. Simulation allows for the viewing of possible dynamic executions of a designed system. In turn, model checking evaluates the exhaustive dynamic behavior of a system. Reachability properties can be verified by exploring the generated state-space of a system.

In order to meet various requirements arising from the diverse application areas, Uppaal has been extended with various features. To comply with different application areas, several versions of Uppaal have been released.

The most well-known version is Uppaal SMC. Since 2012, Uppaal has been extended with statistical model checking [18] to statistically predict valid results regarding system behavior. This kind of verification has great potential, as it allows one to directly evaluate various system models under the same (often stochastic) conditions. Hybrid timed automata models can model deterministic behavior (based on states), non-linear behavior (based on ordinary differential equations), and stochastic behavior, all of them in the same models. Statistical model checking involves several runs of the system with respect to the defined properties. The results obtained are statistical, just to obtain an overall estimate of the design correctness.

Other versions of Uppaal are just as powerful and valuable. Uppaal Stratego [19] is mainly dedicated to strategy analysis. It enables user-friendly performance exploration of different strategies for stochastic timed games before adaptation in a final implementation. Technically, a game is a mathematical model consisting of several players (corresponding to processes) with independent objectives, usually competing, opposing, or even conflicting. Uppaal TIGA [20] aims to solve games. It implements an efficient on-the-fly algorithm for solving games based on timed game automata. Uppaal CORA is focused on cost-optimal reachability analysis. It uses linearly priced timed automata and finds optimal paths (with the lowest cost) to a state satisfying certain goal conditions. Uppaal TRON is a testing tool for the black-box conformance testing of timed systems, suitable for embedded software.

## 3. Research Methodology

### 3.1. Information Sources and Search Strategy

In order to perform this exhaustive review, we searched for the keyword “uppaal” in the titles or abstracts of papers indexed in the scientific databases IEEE Xplore, Elsevier, Springer, ACM, MDPI, and Google Scholar. The search was conducted twice, the first time in September–October 2023 and the second time in January–February 2024, to include also the latest publications. The resulting papers were judged on the basis of abstract scanning. If this was insufficient, a full scan of the article was performed to check whether they were compatible with the inclusion and exclusion criteria.

### 3.2. Research Questions

The research carried out for this review was driven by investigating the main research questions (RQs):

RQ1: What are the application areas of the Uppaal tool?
RQ2: Which version of Uppaal is used the most?
RQ3: Which keywords appear the most often in the obtained papers?
RQ4: What does the distribution of research papers regarding access options, scientific databases, and types of publication look like?
RQ5: What does the distribution of research papers regarding geographical location look like?

In order to identify the current possibilities of the Uppaal tool, we explored the recent literature as the main source to answer these research questions. Based on the research questions, the aim was to present the distribution of publications in terms of application areas, Uppaal versions, access options, scientific databases, and the main research countries. A wordcloud was planned to show the most popular keywords.

### 3.3. Eligibility Criteria

To select only relevant articles that pertained to the topic, some inclusion and exclusion criteria were defined.

The inclusion criteria were specified as follows:

- IC1: Papers published in 2022 and 2023.
- IC2: Research using Uppaal as the main tool.

While criterion IC1 allowed us to narrow down the study to keep it up to date, criterion IC2 limited the articles to those that really focused on the Uppaal tool.

The exclusion criteria were specified as follows:

- EC1: Papers not written in English.
- EC2: Review articles.
- EC3: Papers whose scope was to compare various tools.
- EC4: Papers that could not be evaluated due to very limited access.

Criterion EC1 eliminated papers that were not easily accessible (written in other languages than English). Criterion EC2 omitted review articles that, although they focused on the tool Uppaal, did not use it to achieve specific goals. Criterion EC3 ignored articles that compared various verification tools. Criterion EC4 skipped articles to which there was very restricted access (no open access, limited support for scientific institutions).

### 3.4. Data Extraction, Storage and Analysis

**Figure 1:**
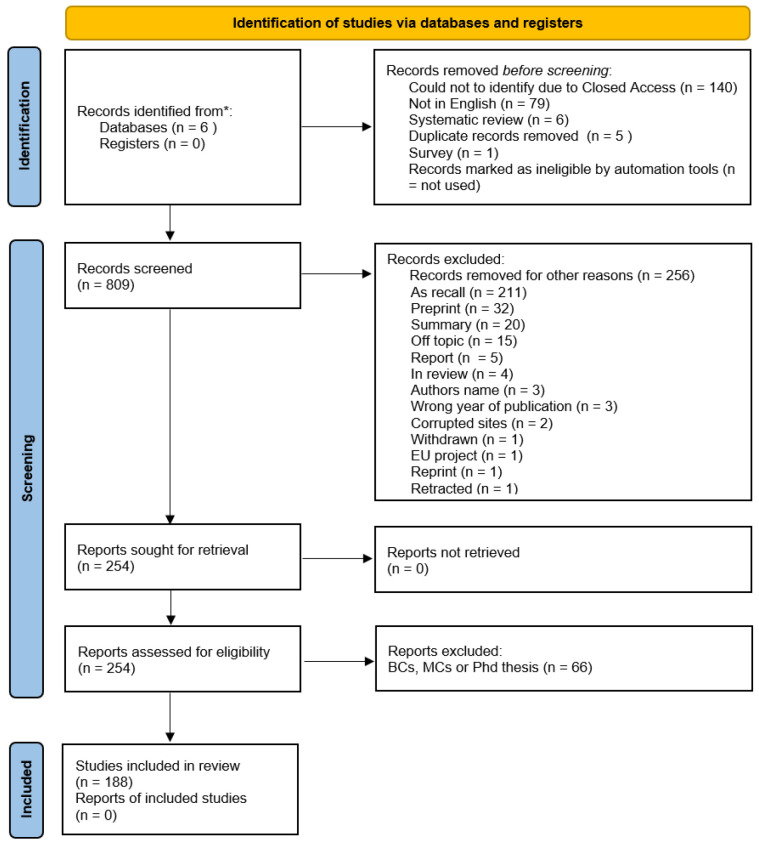
PRISMA flow diagram.

The literature review was performed following the commonly used PRISMA guidelines for new systematic reviews that include database searches. The corresponding PRISMA flow diagram is shown in Figure 1. The initial search in six databases (IEEE Xplore, Elsevier, Springer, ACM, MDPI, Google Scholar) resulted in 1040 papers. It should be noted that some of the papers could not be evaluated because of very restricted access. After applying the inclusion and exclusion criteria, 188 papers were chosen for further detailed analysis.

## 4. Results

This research aimed to find answers to the specified research questions. Let us discuss each of them separately.

### 4.1. RQ1: What Are the Application Areas of the *Uppaal* Tool?

**Figure 2:**
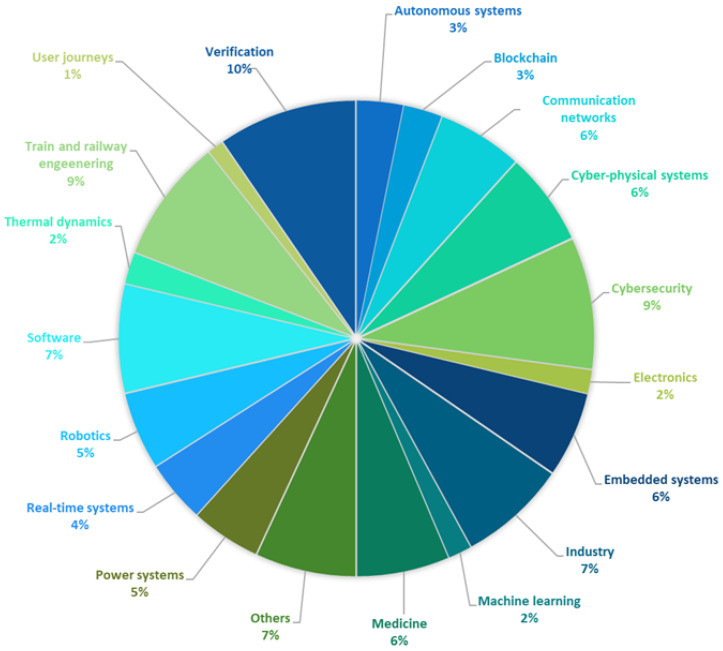
Most popular application areas of Uppaal.

The distribution of publications in terms of application areas is summarized in the pie chart in Figure 2 and is illustrated in more detail in Table 1. It should be noted that not all application areas are mentioned here, only the ones with a number of publications no lower than two. The rest of the articles were classified into the group “Others”.

The distribution of Uppaal applications across various domains highlights its versatility and impact in formal verification. The largest share, 10%, belongs to verification, underscoring the foundational role of Uppaal in model checking, system validation, and schedulability analysis. Close behind are cybersecurity and train and railway engineering (each 9%), reflecting the demand for rigorous safety and reliability standards in these critical domains. Several domains each account for 6–7%, including software, industry, communication networks, cyber–physical systems, embedded systems, and medicine. These areas benefit from modeling timing constraints, concurrency, and uncertainty—particularly in systems where correctness is vital. Power systems and robotics (each 5%) demonstrate significant use in distributed and autonomous environments. Smaller shares appear for real-time systems (4%), autonomous systems (3%), blockchain (3%), and thermal dynamics, as well as electronics and machine learning (each 2%). User journeys (1%) represents a novel application area based on modeling user–service interactions via game-theoretic approaches. The share of 7% attributed to others captures the application of the Uppaal tool in diverse, uncategorized fields.

Let us briefly comment on specific papers and their grouping into the various application domains.

#### 4.1.1. Autonomous Systems

Autonomous systems refer to the types of devices and systems that can operate and perform tasks without human intervention. This domain has been considerably expanding over the years and has become more and more relevant not only in industry but also in daily life. One of the branches of autonomous systems is the autonomous control of vehicles, both terrestrial [24,25,26,27] and aerial [28]. As they become more widespread, the ability to ensure that these systems can handle potential failures (e.g., sensor malfunctions or system breakdowns) is critical. The research papers from this area emphasize that autonomous control has to be thoroughly tested on the design level to ensure the diagnosis and exclusion of any kinds of system failures that could result, for example, in a collision. The usage of Uppaal SMC (e.g., [28]) and Stratego (e.g., [23]) provides the ability to test the constructed formal models and verify their ability to withstand and react to different types of breakage within them and ensure their safety. In [25], the authors find that by using the Uppaal tool, the reliability and realism of virtual testing are enhanced, improving the validity and precision of the testing results.

In the domain of autonomous systems, especially autonomous vehicles, Uppaal offers a powerful framework for the formal verification of complex and time-sensitive behaviors. Studies highlight that formal verification with Uppaal enhances the reliability of such systems. Moreover, it enhances the virtual testing process, making it not only more reliable but also more realistic, which ultimately leads to safer, more robust autonomous systems.

#### 4.1.2. Blockchain

Blockchain is an emerging technology that supports peer-to-peer trade. It records transactions across many computers in a way that ensures both the security and immutability of the exchanged data. As the mentioned aspects are especially important and up-to-date, their verification is significant. The Uppaal tool is used for the runtime monitoring of blockchain executions [29], to evaluate system accuracy without contradictions or errors [30], to verify the framework implementation in smart contracts [31], to check the correctness of smart contracts [32], or for the compliance checking of cloud providers [33].

In the rapidly evolving domain of blockchain technology, ensuring the security and integrity of transactions, smart contracts, and compliance frameworks is essential. The Uppaal tool plays a vital role in verifying these aspects by enabling formal verification for runtime monitoring, system accuracy, smart contract correctness, and cloud compliance. By applying Uppaal to blockchain systems, developers can ensure that blockchain networks and smart contracts are robust, reliable, and secure, which is essential for enabling their widespread adoption and ensuring their trustworthiness in various industries.

#### 4.1.3. Communication Networks

Communication networks enable the exchange and flow of data between individuals. They consist of nodes that communicate with each other. Their existence in daily life enables efficient information flow, especially with fast data transmission. In this area, Uppaal is applied to check deadlock freedom and find worst-case message delivery times for message flows [34]. The Internet of Things (IoT) is a special type of communication network that connects physical devices and other objects with embedded sensors and has recently gained popularity in many applications. The temporal properties of such networks can be verified [35]. Authors have assessed Uppaal as a tool with an intuitive and understandable graphical representation.A self-adaptive IoT system is modeled and verified in [36], in a case study of a smart home with fire detection and an automated lighting system. A Sigfox module for Network Simulator 3 is evaluated in [37]. Another study models and verifies a Sigfox-based IoT network with Uppaal SMC [38]. The lifetime of the nodes is analyzed as a performance metric. Moreover, a set of strategies is evaluated to optimize the battery lifetime of the nodes. An IoT network may be infected with malicious software and then controlled remotely (such an infected network is usually referred to as a botnet). In [39], the dynamic behavior of a Mirai botnet, its infrastructure (used in DDoS attacks), and various categories of IoT devices are modeled and simulated. The possibility of restarting is evaluated as a defense strategy against botnets. The security of IoT networks is also verified in [40]. 5G, as the fifth generation of wireless technology, is also a valid research object. In [41], dynamic service orchestration is modeled and verified. The developed supporting tool does not require any prior experience with timed automata. In [42], a framework is proposed to analyze the RAP (Random-Access Procedure) network protocol with Uppaal and statistical model checking. The Precision Time Protocol for the clock synchronization algorithm in automotive Ethernet is formally modeled in [43]. A new TCP protocol is proposed in [44], with modeling and simulation in Uppaal SMC.

Uppaal has been extensively applied to the modeling and verification of communication networks, including general message flow analysis, deadlock detection, and worst-case delivery time estimation. It is used in IoT systems to verify temporal and security properties, as well as to evaluate some performance metrics, such as node lifetime and energy efficiency. Moreover, the considered studies highlight the versatility of Uppaal in addressing critical aspects of communication networks, particularly in the context of smart home automation, 5G protocols, dynamic service orchestration, or defense strategies against IoT botnets. Usage of the tool can lead to the development of more efficient communication systems, especially in complex, real-time environments such as industrial networks.

#### 4.1.4. Cyber–Physical Systems

Cyber–physical systems (CPSs) integrate computation with physical processes. They are considered to be the core of Industry 4.0. As they usually involve several different technologies, they require more attention than standard software or hardware projects. So, a framework for modeling and analyzing CPSs with the application of SMC is proposed in [45]. SysML is used as a primary specification, while Enhanced Activity Calculus is used for the construction of equivalent-priced timed automata models. Another framework for the design of resilient CPSs with control theory is introduced in [47]. It helps to ensure system stability and safety. A library for the analysis and synthesis of the sampling behavior of event-triggered control systems is presented in [53], with Uppaal Stratego employed for the synthesis of schedulers. Another study, [54], specifies a methodology for the development and security of CPSs. The software in a distributed CPS is formally verified in [48], focusing especially on timing analysis. An intelligent mechatronic component is simulated and validated in [49]. Similar work on the co-simulation of a complex CPS is presented in [50]. Uppaal SMC is used for the validation of strategy switching to improve the fault tolerance of resource-constrained real-time applications [51].

An interesting approach is to use timed games and Uppaal TIGA to determine when an update to a CPS is possible at certain run-time [46]. Up-to-date practical research deals with the Digital Twin (model replica of a physical system) setup for safety-aware optimization, which is verified in Uppaal [52]. The tool also helps identify potential threats to CPSs [55]. Cyber bio-analytical physical systems are designed in [56], with Uppaal Stratego employed for the analysis of the interaction of several devices.

Due to the multidisciplinary nature and complex interaction patterns of cyber–physical systems, they demand rigorous design methodologies beyond those employed in conventional software or hardware systems. A diverse range of formal approaches have been proposed to support modeling, verification, and synthesis, with a strong emphasis on correctness, safety, and resilience. Applications range from secure CPS development and timing verification in distributed systems to simulation, co-simulation, and Digital Twin implementations. A notable trend involves extending CPS modeling to domains such as cyber bio-analytical systems. The considered studies reveal a common focus on early-stage verification, real-time constraints, and adaptive control under uncertainty. Most recent approaches focus on modeling, proposing either a new methodology or even a whole framework.

#### 4.1.5. Cybersecurity

Cybersecurity usually refers to protecting systems, networks, or programs (in general, hardware and software) from digital attacks. It is constantly being updated due to increasing threats. In [57], a new formalism for the defense of moving targets is proposed, and the attack time and cost distributions are calculated under various attacker strategies. The authors use Uppaal Stratego, although they consider implementing their own tool to find the best strategies. A user-specific security policy is generated through the formal modeling of user behavior in [58]. The authors show how to identify and select the essential characteristics that define user security behavior. The identified behaviors are modeled for the purpose of automated reasoning. This allows weaknesses to be found in users’ security behavior and enables the proposal of some relevant policies.

In [59] a modeling and analysis method for industrial control system functions is proposed to ensure that supervisors can work properly under potential cyberattacks. Similar work by the same authors, this time with a resilient third-party monitoring system, is also presented in [62]. The security verification of cyber–physical systems is also addressed in [60], where the authors find that exploring the human–machine interaction requires performing an exhaustive search for each state in all combinations of feasible models. Distributed Denial of Service (DDoS) is formally specified in [61]. The functional requirements of the protocol are verified in order to determine the accuracy of the system. The attack resistance of a controller area network system (CAN) is enhanced in [63]. A comprehensive model combining a variable attacker with a CAN bus is proposed, and Uppaal SMC is applied to determine the statistical probability of transmission and response behavior of the CAN bus. An effective property-checking method and a formal verification framework for hardware Trojan detection are proposed in [64]. The model-based risk analysis framework of the Attack–Fault Maintenance Tree is verified in [65], with some statistically valid safety/security metrics, and the impact of coordinated cyber–physical attacks is analyzed in [66]. The authors state that statistical model checking allows better scaling of the system dimension under security analysis. Moreover, the modeling language provided by Uppaal SMC has proven to be effective in specifying realistic systems.

A three-side-channel attack is analyzed in [69], with probabilistic hyper-property logic for stochastic hybrid and timed systems. Safety violations are effectively detected through randomized reachability analysis in [67]. The work is published with the affiliation of Aalborg University, Denmark, and additionally provides some implementation details regarding the Uppaal tool (with a reduction in the checking time of some properties from 23 h to 23 s). An Uppaal execution engine and Uppaal TRON were used for the development of an MUPPAAL tool [68] that allows mutation testing (artificial faults are injected into the system and the ability of tests to distinguish these mutants is evaluated). A SIM box fraud prevention system utilizing fingerprint-based access policies is analyzed in [70], with a detailed security analysis of the access control list. An ontology-based framework for formal verification of the safety and security properties of control logic is introduced in [71]. Model-driven software development with the analysis of safety and security properties is discussed in [72], where the authors point out that the time-consuming process of model checking may disqualify this technique among programmers. Voting protocols are the subject of [73], where the authors try to make security measurable.

Uppaal and its extensions have been successfully applied in the field of cybersecurity to model, simulate, and verify a wide range of security-critical systems. These include the formal analysis of attack–defense strategies, the generation of user-specific security policies, and the verification of system resilience under cyberattacks in industrial control systems. Specific applications include the modeling of DDoS attacks, the enhancement of CAN bus security, the and verification of hardware Trojan detection mechanisms. Statistical model checking has proven particularly useful for evaluating system behavior under uncertainty and scaling to large system dimensions. Further contributions include the analysis of side-channel attacks, randomized reachability for safety violation detection, and mutation testing for software robustness. Researchers have also explored formal frameworks for SIM box fraud prevention, the ontology-based verification of control logic, and secure model-driven software development. While formal verification using Uppaal offers strong guarantees, some studies highlight the trade-off in verification time, which may hinder its widespread adoption among developers.

#### 4.1.6. Electronics

This narrow specialization also benefits from the Uppaal tool. In [74], the logic of the processor’s behavior-level code is analyzed. Among others, deadlock freedom is confirmed. In [75], an embedded pulse-transfer-level language for superconductor electronics is proposed, together with a framework, and verified with Uppaal. Multicore processors and interference analysis are the subject of [76], where Uppaal is used to compute an upper bound of the number of interferences experienced by each task in each component for each segment.

In the domain of electronics, simulation-based verification remains dominant due to its scalability and widespread tool support, while formal verification, including model checking, is used more selectively for verifying complex control logic, timing correctness, and safety-critical properties in electronics. The considered studies confirm that they benefit from using Uppaal through its capability to model and verify low-level hardware behavior and timing-critical systems.

#### 4.1.7. Embedded Systems

Embedded systems combine hardware and software and are designed for a specific function. The feasibility of using ciphers in an embedded real-time operating system (RTOS) is investigated in [77]. An operating system architecture for sustainable embedded systems is proposed in [78], where application tasks are modeled with Uppaal (incorporated into networks) and different aspects of (non)functional properties are verified. In [79], a modeling concept for the formal verification of compositional software based on an operating system is introduced, with an RTOS kernel used as the modular model, and verified in terms of task synchronization and resource management timing. In [80], a time-related model checking approach is proposed for the specification of software requirements in embedded systems to show possible software behaviors. The safety-critical embedded systems of avionics are evaluated in [81].

A middleware that supports the development of embedded multi-agent systems to prevent a lack of connectivity is presented in [82], with formal modeling and verification performed using the Uppaal tool. Various communication methods are evaluated. Model checking is combined with reinforcement learning to solve the multi-agent autonomous system planning problem in [83]. A new method called MoCRel is integrated into Uppaal Stratego, and the experiments show that it can solve the planning problem in complex maps with large numbers of agents performing various types of tasks. Similar work on path planning and task scheduling strategies for multiple autonomous agents [84] resulted in the integration of the improved MCRL method into Uppaal Stratego. An intuitive agent-based abstraction scheme is studied in [85], with a reduction in state space achieved. The correctness of the approach is formally proven. A multi-agent reasoning-based context-aware model is proposed in [86], with formal verification of the correctness properties achieved. The liveness and real-time requirements of OS-based embedded software are analyzed in [87]. The limited number of properties is considered; nevertheless, the proposed modeling strategy is said to be scalable.

The analyzed studies show that Uppaal has been widely utilized in the formal verification of embedded systems, enabling the analysis of real-time properties, task scheduling, and resource management in RTOS-based architectures. Its application is extended to safety-critical embedded systems and multi-agent systems, where integration with methods like MoCRel and MCRL (for Uppaal Stratego) facilitates scalable planning and verification. These studies demonstrate the effectiveness of the tool in ensuring correctness and good performance in complex, time-sensitive embedded environments.

#### 4.1.8. Industry

All pure industrial approaches have been included in this category. An industrial control network protocol is modeled and its reliability is verified in [88]. Collaborative manufacturing is modeled and the production cost is simulated in [89]. An intelligent product line manufacturing system is verified in [90]. In [91], a twin-based digital automatic programming method is introduced for the adaptive control of manufacturing cells using the simulation feature of Uppaal. A complete process for assessing the robustness of schedule solutions is proposed in [93], supported by Uppaal SMC. A reconfigurable fault-tolerant control framework applied to a manufacturing system is presented in [94], and is verified before implementation with Uppaal. The safety of multiple industrial robot manipulators with path conflicts is verified in [92]. Verification of the human-adapted programmable logic controller (PLC) code, according to the IEC 61131-3 standard, is discussed in [95]. An ontology-based framework for industrial control systems is introduced in [96]. A similar automated tool-supported quantitative risk analysis framework is also proposed in [97], using Uppaal SMC for simulations. A Brake-by-Wire industrial prototype system that improves road safety is used as a case study in [98]. An industrial manufacturing control system is extended with system defenses in [99], providing some recommendations for the development of future defensive strategies. The construction industry is the research area of focus in [100], where the authors conduct formal modeling and verification of the credibility of knowledge. An analysis of the scalability and performance of a persistent storage approach is performed in [101], where fault tolerance and data consistency are verified with Uppaal.

In industrial systems, Uppaal has been extensively applied to model, simulate, and verify control logic, system robustness, robotic manipulators, and real-time constraints across various manufacturing and automation scenarios. Its applications include the verification of industrial protocols, cost-aware scheduling, fault-tolerant control frameworks, and collaborative manufacturing systems. An interesting approach is the validation of human-adapted PLC code in compliance with IEC standards. Case studies such as Brake-by-Wire systems and persistent storage solutions demonstrate the capacity of Uppaal to analyze safety, scalability, and fault tolerance. These contributions underscore that Uppaal is valuable in enhancing reliability and security in complex industrial automation environments.

#### 4.1.9. Machine Learning

Machine learning is considered a type of artificial intelligence (AI) in which computers learn from the provided data and improve with experience. In [102], the partitioning-refinement learning method of Uppaal Stratego reduces the expected number of guesses in the popular Wordle game by almost half. In [103], a technique is proposed to learn explainable timed automata from passive observations of a black-box function. A black-box function may be an artificial intelligence system. A prototype was implemented and evaluated by learning two controllers, namely a brick-sorting conveyor belt trained with reinforcement learning and a real-world derived smart traffic light controller. A novel approach to analyze large adaptation spaces is proposed in [104]. Using classic supervised machine learning techniques to reduce adaptation spaces on the fly is suggested. The analysis of the adaptation options is performed with Uppaal SMC.

Uppaal, particularly the Stratego and SMC versions, has been applied to integrate formal verification with machine learning techniques. It supports learning optimal strategies, as demonstrated in strategy synthesis for games like Wordle, and in explainable model inference from black-box systems using timed automata. Its applications include reinforcement learning-trained controllers, real-world systems such as traffic lights and sorting conveyors, and the analysis of large adaptation spaces by incorporating supervised learning to reduce complexity during system adaptation.

#### 4.1.10. Medicine

Medicine is a branch that is very important for entire populations around the world. Technological progress offers new possibilities for better treatment of patients, but at the same time, any new solution must be fully reliable to ensure people’s safety. A medical resource utilization process is modeled in [105]. Afterwards, it is verified with Uppaal to ensure that all safety requirements are met. A healthcare system Serums is introduced in [106], where formal methods are used to verify its safety and security requirements. The quality of service of a healthcare system is ensured with Uppaal in [107], especially focusing on reliability and security. In a runtime environment, a clinically interpretable classification of arrhythmias is verified in [108]. The efficacy of the approach is evaluated in conjunction with existing clinical ECG databases. A healthcare application based on the Internet of Things is formally analyzed in [109], considering mainly the properties of safety, liveness, and deadlock freedom. A unified healthcare communication system is analyzed against threats in [110]. Uppaal SMC is used to detect the most probable type of attacks resulting in the mistreatment of patients. In turn, a telerehabilitation system is formally verified in [111] that also promotes formal methods for the design of safe medical software systems.

Special attention has been paid to specific illnesses. Deep brain stimulation controllers for Parkinson’s disease treatment are investigated in [112], and Alzheimer’s disease is the research subject in [113], while prevention strategies for COVID-19 exposure are analyzed in [114] (using Uppaal Stratego). The functional prototype of a mechanical ventilator is verified and improved in [115]. A bioelectronic system connecting medical devices and a biological system is verified in [116] to check properties such as the reachability of hazard-related states.

Medicine is a critical domain where technological innovations must meet the highest standards of safety and reliability. In this context, Uppaal has been successfully employed to formally verify a wide range of healthcare applications, ensuring adherence to essential safety, security, and liveness properties. These include medical resource utilization processes, IoT-based healthcare systems, quality of service in healthcare infrastructures, and clinically relevant models such as arrhythmia classification, Alzheimer’s models, or Covid-19 exposure prevention strategies. The considered studies demonstrate how formal verification contributes to the development of dependable and secure medical systems.

#### 4.1.11. Power Systems

Power systems consist of electrical components and are used to supply and transfer electrical power. The performance of the predictive control algorithms of a Finite Set model applied to a matrix converter is statistically verified in [117,125]. The results obtained can be used to extend the lifetime of these devices during various grid conditions. Protection systems in low-voltage distribution grids are formally verified in [118,123]. Power smart IoT entity services are modeled in [119] in order to increase their feasibility and stability. A new mobility- and energy-harvesting-aware medium-access control protocol is modeled in [120]. Its performance is evaluated with Uppaal SMC. A control strategy for a battery management system is checked with Uppaal Stratego in [121]. A case study of a research nuclear reactor is presented in [122]. Here, Uppaal Stratego is used to find the number of spares that minimizes the total costs of downtime and purchase of spares, all in a short period of time. The flexible behavior of energy systems to balance the production and consumption of energy is modeled and analyzed in [124].

Power systems increasingly rely on formal verification to ensure efficiency, safety, and adaptability. Uppaal and its extensions (SMC and Stratego) have been applied to verify control strategies for converters, battery systems, and grid protection mechanisms. It also supports the modeling of energy-aware IoT services, communication protocols, and flexible energy balancing. These applications demonstrate that Uppaal is able to support strategic decision-making in modern energy infrastructures.

#### 4.1.12. Real-Time Systems

Real-time systems are supposed to respond within a specific time period to incoming inputs and commands. The schedulability of an acquisition–execution–restitution task model is analyzed in [126]. Embedded real-time systems are verified in [127], checking, e.g., the bounded response. Execution strategies for temporal networks with various sources of uncertainty are computed in [128] with the modeling of possible reaction times (using Uppaal TIGA). The time-sensitive software-defined network architecture is verified in [129], among others, against deadlock freedom and starvation freedom. A framework for the quantitative evaluation of cyber–physical–social system performance is modeled and verified with Uppaal SMC in [130]. Equivalent mutants in real-time model-based mutation testing are detected with Uppaal TIGA in [131]. The same version of the tool is used in [132] to synthesize safe controllers for continuous-time sampled switched systems. Event logs in real-time systems are investigated in [133] with classical Uppaal and Uppaal SMC.

Real-time systems require strict timing guarantees, and Uppaal—along with its TIGA and SMC extensions—has proven to be effective for their formal verification. It is applied to analyze task schedulability, bounded response times, execution strategies under uncertainty, and time-sensitive network properties; for detecting equivalent mutants in testing; and for synthesizing safe controllers. These applications show that Uppaal is suitable for verifying the correctness and reliability of time-critical systems.

#### 4.1.13. Robotics

Robotics is a branch of engineering focused on the design, creation, and operation of robots that can perform their tasks automatically. In [134], multi-robot interactive scenarios in service settings are formally modeled and verified with Uppaal SMC. Interactive robot service applications are also the topic of [135], where a model-driven framework integrated with Uppaal SMC is presented. Real-time autonomous robots are formally verified in [136], with several important requirements checked. Dynamic route planning for a fleet of autonomous mobile robots is discussed in [137] with the application of Uppaal Stratego. The correct behavior of multi-robot autonomous systems is ensured in [138], where a framework is introduced that integrates design and formal verification at a higher level of abstraction. The impact of human error on interactive service robotics scenarios is analyzed in [139]. Latencies and buffer overflow in distributed robotic systems are investigated in [140]. The work highlights the advantages of Uppaal and indicates that it can reveal potential errors that are not detected by experiments. Service robots with uncertain human behavior are considered in [141], with the proposal of a framework built on formal modeling, verification, and learning techniques. Explainable service robots and their software architecture are addressed in [142], with a formal analysis conducted using Uppaal SMC. In [143], a decentralized solution for high-level multi-agent task planning problems is proposed, with Uppaal used to synthesize a plan that provably satisfies the updated task.

Robotics benefits significantly from formal verification using Uppaal and its SMC and Stratego extensions, in particular, to verify multi-robot systems, autonomous navigation, and real-time service applications. Key contributions include route planning, task coordination, handling uncertain human behavior, and evaluating performance issues like latency and buffer overflows. So far, Uppaal has been proven to enhance reliability in the domain of robotics, especially in human-interactive and real-time systems.

#### 4.1.14. Software

Software engineering aims to create reliable, efficient, and scalable applications that we use everyday. Additional verification evidently contributes to an increase in quality. As history shows, software failures can be expensive and have catastrophic consequences, as was the case with Ariane 5. In 1996, the flight ended in failure, as 40 s after initiation, the launcher broke apart and exploded. The Inquiry Board report can be found at https://www.esa.int/Newsroom/Press_Releases/Ariane_501_-_Presentation_of_Inquiry_Board_report (last accessed on 14 February 2025). In this context, the authors of [144] validate RabbitMQ—an implementation of the Advanced Message Queuing Protocol. The basic properties are checked, for example, data reachability or data concurrency. An open-source business process management system, YAWL, is verified in [145]. An automata-based approach to manage self-adaptive component-based architecture is proposed in [146], where the consistency of the software is checked before the adaptation implementation. In [147], a new runtime environment is introduced for the coordination of services in contract-based applications. The absence of deadlock was verified with Uppaal. The time behavior of self-adaptive software under uncertainty is modeled and analyzed in [148], and the probabilistic behaviors of the model are verified with Uppaal SMC. Distributed shared-memory algorithms are formally checked in [149], with the authors employing problems related to state explosion.Context-oriented chatbot conversational flows are modeled in [150], allowing one to overcome some verification gaps that are not able to be overcome via other testing techniques. Colluder detection in SaaS (software as a service) cloud applications with subscription-based licenses is the subject of [151]. An integrated co-simulation and synthesis framework for the stochastic model-predictive control of software controllers (called STOMPC) is proposed in [152], with Uppaal Stratego employed as the engine. It is said to be generally applicable across different application domains, including traffic light control or building floor heating. In [153], it is used for synthesizing safe and near-optimal control strategies for stormwater detention ponds. Self-adaptive systems are the subject of [154], where Uppaal SMC is used to verify adaptation options. Similarly, reconfiguration strategies for self-adaptive systems are precomputed with Uppaal Stratego in [155]. Near-optimal solutions are approximated. Another approach for engineering self-adaptive systems is proposed in [156] with the application of Uppaal SMC. The real-time behavior of an application for tennis training is verified in [157] to ensure that the safety requirements are met.

Uppaal plays a critical role in improving software reliability, particularly in systems where failures can have catastrophic effects. It has been used to verify messaging systems (e.g., RabbitMQ) and business process tools (YAWL). Uppaal SMC has been successfully applied for analyzing probabilistic behaviors in adaptive systems, runtime environments, and chatbot interactions, as well as in co-simulation frameworks for traffic and building control. In turn, Uppaal Stratego has been applied to synthesize near-optimal adaptation strategies and control policies in contexts such as SaaS license enforcement, stormwater detention systems, and self-reconfigurable software systems.

#### 4.1.15. Thermal Dynamics

Thermal dynamics is gaining importance with the growing popularity of sustainability, eco-friendliness, and energy-saving policies. A toolchain for controlling a domestic heat pump in a floor heating system is proposed in [158,160]. The predictive model is prepared and evaluated with the use of Uppaal Stratego. The same version of Uppaal is applied for residential heat pumps with uncertain weather forecasts in [159]. The minimum and maximum flexibility potentials of the pumps in optimistic and pessimistic energy consumption patterns are calculated, and the impact of weather forecast on the flexibility of heat pumps is investigated. A similar research topic, namely the thermal dynamics of residential buildings with energy flexibility, is addressed in [161], where the heat-to-power flexibility of heat pumps is evaluated.

Thermal dynamics benefits from using Uppaal, in particular, its Stratego extension, for optimizing heat pump control in residential heating systems. Predictive models have been synthesized and evaluated under uncertain weather conditions to assess flexibility potential. Studies show how forecast variability impacts energy flexibility and the heat-to-power adaptability of domestic heat pumps.

#### 4.1.16. Train and Railway Engineering

Train and railway engineering focuses on the development and maintenance of modern railway infrastructure. Railways are one of the most popular and energy-efficient ways to transport both humans and cargo. Moreover, in many countries they are considered to be part of critical infrastructure. Due to these facts, it is essential to provide the highest standards of safety and perform meticulous verification of all components, i.e., systems [164,167], algorithms, and communication protocols [163], before their implementation to ensure reliability and minimize the risks of accidents. In [162], a formally verified scheme is proposed to manage train communication information. The application of a Unified Modeling Language (UML) supporting railway engineers, together with Uppaal, is shown in [165]. A risk evaluation method for autonomous trains is proposed in [166]. A description of the usage of Uppaal for the formal verification of the ERTMS (European Rail Traffic Management System)/ECTS (European Train Control System) can be found in [168,169,171,173]. A novel approach for designing electronic urban trains via model verification is presented in [170]. A movement authority scenario in a train-centric communication-based train control system is analyzed to determine its safety in [172] with Uppaal SMC. Urban rail transit is the topic of [174], where the authors conduct formal verification of security requirements. Models of a safety-critical motor controller in railway systems are evaluated in [175]. An interesting discussion on research into formal methods that can contribute to the development of modern railway systems is presented in [176]. Future train control systems are also considered in [177].

Train and railway engineering increasingly relies on formal verification. Uppaal is widely applied to validate railway systems, algorithms, and communication protocols, and ensure compliance with safety standards. Its specific applications include ERTMS/ETCS verification, autonomous and urban train systems, risk evaluation, and movement authority scenarios. Model-driven approaches and UML integration further support reliable system development in this safety-critical sector.

#### 4.1.17. User Journeys

A separate category is dedicated to user journeys. This refers to the process a user goes through when interacting with a service or a system. User journeys are formalized as weighted games (user versus service provider) in [178]. Uppaal Stratego is used to discover challenges in the interaction between customers and a company. The other focus of their research is on multiparty event logs (an extension of event logs with information on the parties) [179] that allow the analysis of user journeys.

By treating user journeys as strategic games or enriched event logs, Uppaal Stratego enables the precise identification of interaction challenges and behavioral insights, supporting the design of more reliable and user-centric systems.

#### 4.1.18. Verification

In general, the key application of the Uppaal tool is the exact verification of various kinds of systems. In this category, we have classified works that contribute considerably to verification methods, although they could also be considered to fit into one of the other categories. In [180], a tool is proposed that aims to integrate formal analysis and the verification of functional requirements. The robustness of timed automata is analyzed in [181]. Uppaal is used here for sufficiency checks and for computing witnesses of the proposed methods.

Stochastic time automata and proof of their correctness are the subject of [182]. The zone-based verification of timed automata is considered in [183]. Hardware/software co-design with Uppaal SMC is addressed in [184], going from UML MARTE (providing foundations for model-based descriptions of real-time and embedded systems) specification to early functional verification. The practical aspects of test automation with efficient test models developed by the authors are considered in [185], with the aim of reducing the effort required to create models. Compliance through model checking is addressed in [186]. Model-based testing is combined with automated analysis in [187], focusing especially on reachability and deadlock freedom properties. An approach to constructing a target clock state in a model with sequences of difference-bound matrix operations is proposed in [188]. In [189], a learning-based framework is introduced for assume/guarantee reasoning.

The use of Monte Carlo Tree Search for model checking is evaluated in [190], with the experiments performed in Uppaal CORA. A rare event simulation technique is incorporated into Uppaal SMC in [191]. A property specification pattern catalog is proposed in [192], allowing practitioners to specify qualitative requirements based on patterns (eliminating the use of temporal logic). Aspect-oriented modeling, where correctness is ensured by the construction, is presented in [193]. Temporal modalities to extend the notion of assume/guarantee contracts are introduced in [194], focusing on practical aspects of test automation. The diverse aspects of modeling and quality assessment are discussed in [195]. Dynamic timed automata for the modeling and verification of reconfigurable systems are proposed in [196], and are transformed into semantic equivalent timed automata in Uppaal format. An open-source tool, Uppex, is described in [197], which automatizes feature analysis by combining Microsoft Excel spreadsheets and Uppaal models. This allows the authors to reach the right balance in the level of details—enough detail to be trustworthy but not so much that it hinders the verification of complex requirements.

The Uppaal tool plays a central role in the formal verification of diverse system models, with numerous studies advancing its methodologies beyond domain-specific applications. Key theoretical developments include the robustness analysis of timed and stochastic automata, zone-based verification techniques, and assume/guarantee reasoning frameworks. Complementary to these are practical contributions aimed at improving the efficiency and accessibility of verification, such as model-based testing, compliance verification, and automated test generation. The considered studies demonstrate the rich versatility of Uppaal and its sustained relevance in advancing both the theory and practice of system verification.

#### 4.1.19. Others

In this category, we have placed articles that do not strictly match the particular application areas considered above. Nevertheless, it should be noted that this is only our subjective opinion, and some aspects relevant to different domains can also be identified in these papers.

Stochastic Reward Nets (a subtype of Petri nets) are formally reduced and analyzed in [198]. A novel modeling and analysis approach is proposed, aimed at checking model correctness. UML state machines are translated into timed automata in [199] using Uppaal semantics. Twin clutch gear control to support drivers is formally verified in [200], and the results indicate that the model partially meets its functional requirements. The application of Uppaal SMC to comply with the safety and efficiency control laws of multi-car elevator systems is investigated in [201].

In [202], a gossip-based information dissemination protocol is introduced to improve distributed system resiliency, where client–server systems are modeled and analyzed with Uppaal SMC. Similar work by the same authors [203] evaluates the efficacy of the proposed approaches in improving the relative performance of three models. Asynchronous systems with a timed integrated model of distributed systems are successfully modeled and verified in [204] for small, medium-sized, and large system models. Digital Twins are addressed in [205], where their foundation model is formally verified in Uppaal. A methodology and tool, called A2A, that automatically models systems defined by the Autosar specifications as timed automata is proposed in [206]. The timing properties of the model are then verified using Uppaal. The clock synchronization algorithm of an in-vehicle network, FlexRay, is formally modeled and verified in [207]. An approach to building better trust in human–machine teaming by combining model checking and machine learning is presented in [208] and verified with Uppaal SMC.

The behavior of an unmanned aerial vehicle (UAV) cluster is modeled in [209], and the authors perform formal verification of a cluster attack mission. In [210], an assume/guarantee framework for additive compositional reasoning in the setting of hybrid systems is presented. The authors show how Uppaal SMC may be used to efficiently falsify refinements.

This category encompasses diverse applications of the Uppaal tool not classified under the other domains listed above, demonstrating the versatility of Uppaal in modeling and verification. Several works explore foundational modeling transformations, such as translating UML state machines into timed automata or reducing Stochastic Reward Nets to verify correctness. Uppaal’s Applications range from verifying control logic in automotive systems to improving the safety and efficiency of digital infrastructure. Notably, Uppaal SMC is employed in research focused on large-scale asynchronous and distributed systems, showcasing its scalability and robustness. Emerging domains like Digital Twins and UAV clusters also benefit from formal verification. These contributions highlight the adaptability of Uppaal across novel and complex system architectures, reinforcing its role as a versatile tool in formal verification research.

### 4.2. RQ2: Which Version of *Uppaal* Is Used the Most?

**Figure 3:**
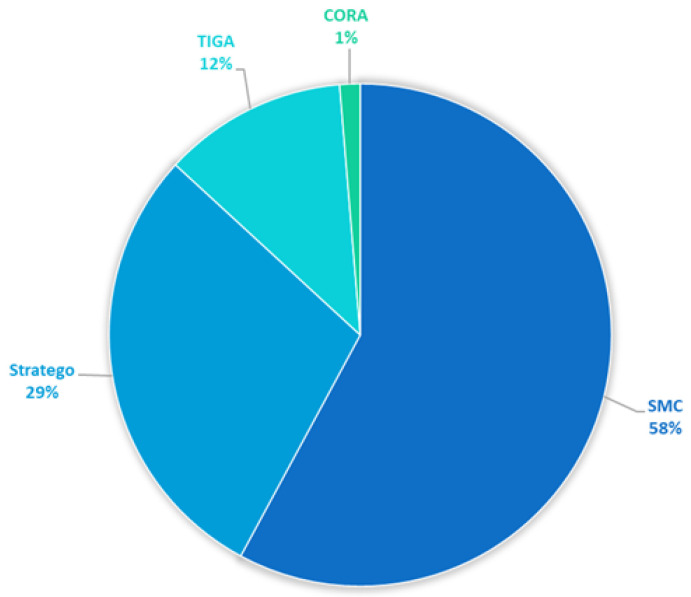
Uppaal versions (explicitly mentioned in 76 papers).

The distribution of publications in terms of the Uppaal version is summarized in the graph in Figure 3. It should be noted that the results are based on the analysis of papers that explicitly reported the tool version (n = 76). As can be seen in the diagram, Uppaal SMC dominates the landscape, accounting for 58% of publications. This prevalence underscores its growing relevance in contemporary system design, particularly for applications requiring stochastic modeling, performance evaluation, and probabilistic verification. It also indicates an important future research direction. Uppaal Stratego, used in 29% of the studies, reflects the increasing interest in synthesis and strategy optimization under uncertainty. Uppaal TIGA accounts for 12% of the total share, highlighting its niche application in controller synthesis within timed games. Uppaal CORA is used occasionally (referenced in only 1% of the papers), indicating limited but focused usage, likely due to its specialization in cost-optimal reachability analysis. The prominence of the SMC and Stratego extensions suggests a research shift toward quantitative analysis and automated strategy generation, aligning with trends in cyber–physical systems and adaptive control.

### 4.3. RQ3: Which Keywords Appear the Most Often in the Obtained Papers?

**Figure 4:**
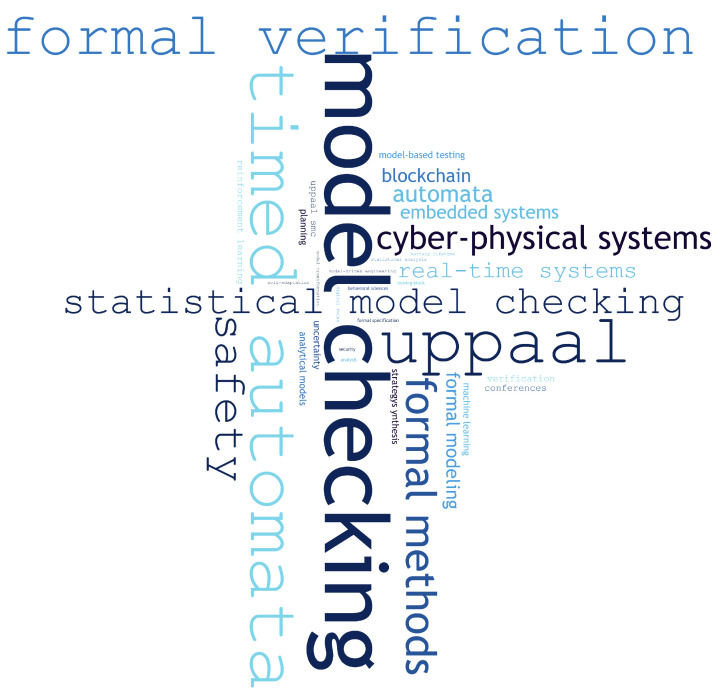
Wordcloud with keywords appearing in the obtained papers.

During this study, we collected the keywords that occurred in the papers that used Uppaal. Their frequency of use is illustrated in Figure 4, where the larger the font, the more often a given word appeared. As expected, “model checking” emerges as the most prominent term, emphasizing the central role of Uppaal in formal verification processes. Other frequently occurring terms include “timed automata” and “formal verification”, which highlight the tool’s foundational basis in timed models and its application in rigorous system correctness analysis. The presence of terms such as “cyber-physical systems”, “safety”, and “real-time systems” further reflects the widespread use of Uppaal in verifying critical timing and reliability requirements in modern system design. The keyword distribution confirms strong alignment of Uppaal with contemporary challenges in verifying complex, time-sensitive, and safety-critical systems.

**Figure 5:**
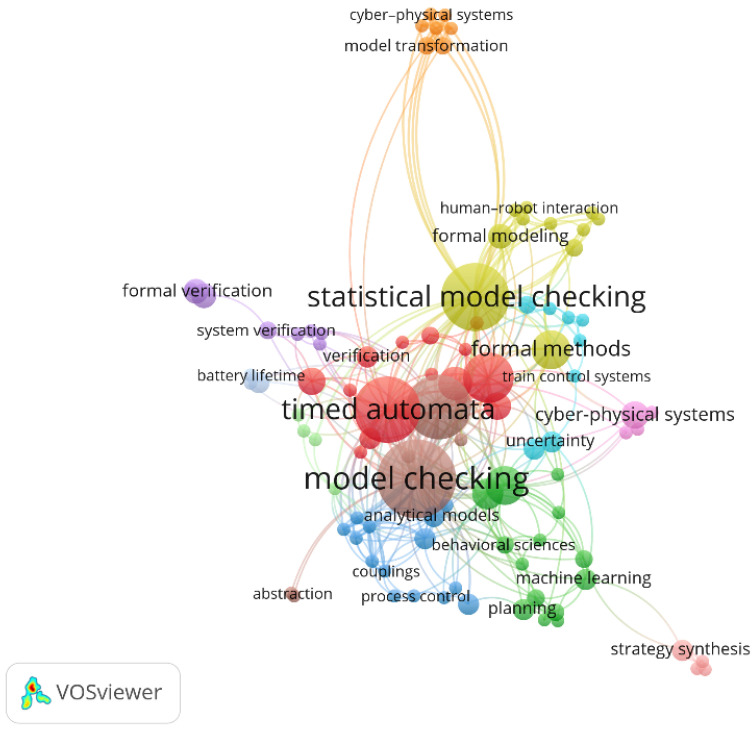
Keyword co-occurrence network visualization.

Additionally, we prepared a keyword co-occurrence network visualization, shown in Figure 5. Each node represents a keyword, while the edges indicate co-occurrence links across the analyzed publications. The size of the nodes, similarly to in a wordcloud, reflects the frequency of keyword appearances, and their proximity and clustering suggest thematic affinities. The largest and most central nodes—“model checking”, “timed automata”, and “statistical model checking”—form the core of the network, reaffirming their foundational role. Surrounding these central terms are several interconnected clusters, each representing a distinct thematic focus. For instance, the dark green cluster includes keywords such as “machine learning”, “planning”, and “behavioral sciences”, reflecting the growing interest in integrating learning-based approaches with formal verification. The light green cluster centers around “formal modeling”, “human-robot interaction”, and “cyber-physical systems”, indicating the relevance of Uppaal in human-centered applications. Smaller, more specialized clusters, like the blue group focused on “analytical models”, “couplings”, and “process control”, highlight niche application areas. The presence of bridging terms such as “formal methods” and “verification” illustrates the tool’s interdisciplinary applicability and its integration into diverse system analysis workflows.

**Figure 6:**
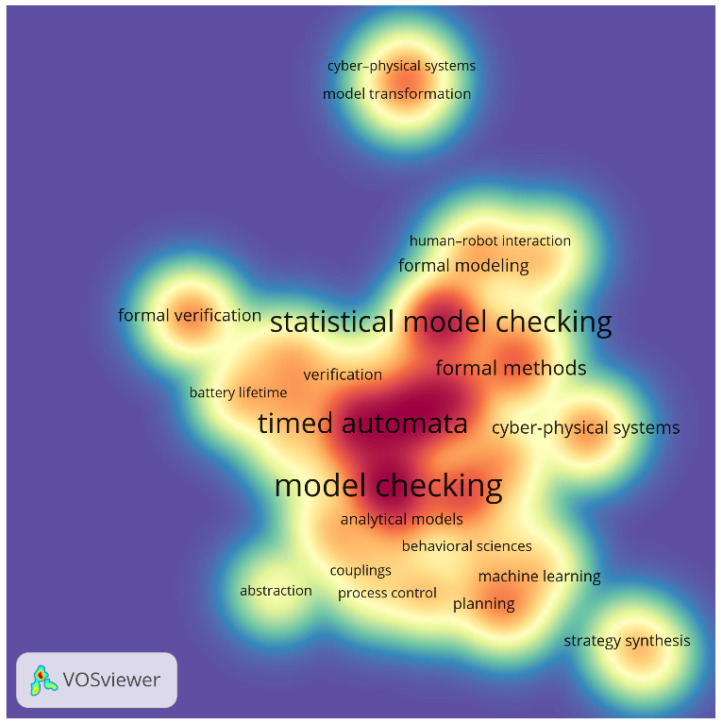
Keyword density visualization map.

Another visualization, a keyword density visualization map, is shown in Figure 6. Unlike the co-occurrence network, which emphasizes thematic relationships, the heatmap offers an indication of the prominence of a given keyword within the research landscape. It complements the structural view by offering quantitative visual cues about topic saturation and marginality. The brightest zones reflect strong scholarly focus, revealing where research efforts have been most concentrated. The red region underscores the core methodological focus of the field, while more diffusely colored areas suggest emerging or less-explored niches. Unsurprisingly, the densest areas center around “model checking”, “timed automata”, and “statistical model checking”, confirming their foundational role in the field. Interestingly, the visible separation of dense regions implies that although some topics are conceptually linked, they are investigated with varying degrees of emphasis, pointing to opportunities for interdisciplinary integration or underexploited research directions. Moderate density is observed in the areas linked to “machine learning”, “formal methods”, and “formal modeling”, suggesting growing intersections between classical verification techniques and data-driven or human-centered approaches. In contrast, peripheral topics such as “strategy synthesis” or “human-robot interaction” appear in cooler zones, indicating that while they are connected, they remain niche or less frequently studied within this domain.

### 4.4. RQ4: What Does the Distribution of Research Papers Regarding Access Options, Scientific Databases, and Types of Publication Look Like?

**Figure 7:**
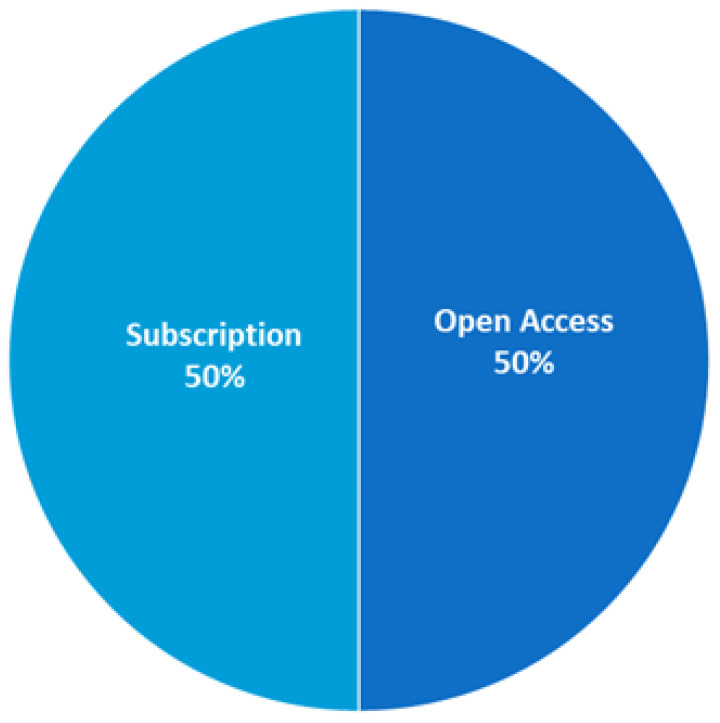
Types of license.

It was interesting to learn which access option the authors of the papers chose, which scientific databases they chose, and which types of papers they chose to present the results of their research. The distribution of publications in terms of access options is summarized in Figure 7. In line with the global trend of making research results available for a wide range of readers, the proportion of open access articles is significant and currently balances the number of non-open access articles. This parity aligns with the broader trend toward open science, reflecting the growing emphasis on research transparency, accessibility, and public engagement. On the other hand, the continued presence of subscription-based articles indicates that traditional publishing venues still retain influence, particularly in well-established, peer-reviewed journals.

**Figure 8:**
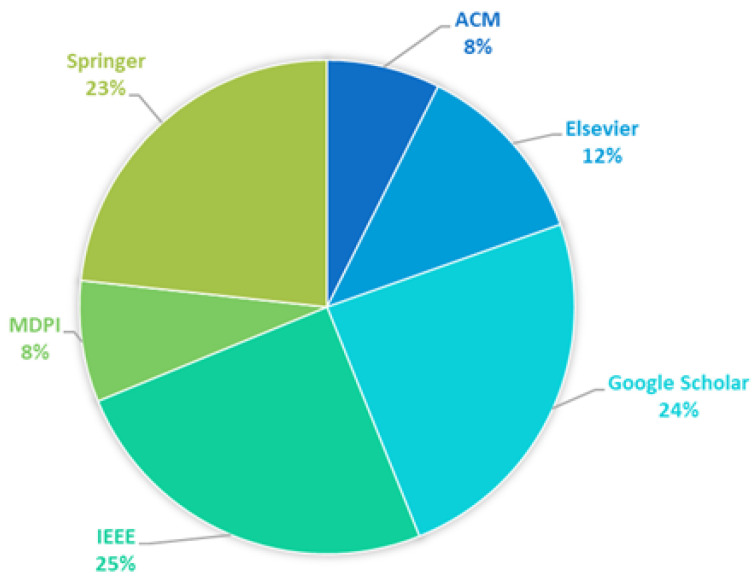
Scientific databases.

The distribution of publications in terms of the indexing database is summarized in the chart in Figure 8. The three leading scientific databases achieve comparable results and together account for almost 75% of all considered works (IEEE Xplore (25%), Google Scholar (24%), and Springer (23%)). These platforms are widely recognized for their broad visibility. In contrast, the other databases have a much smaller yet notable share (slightly more than 25% of papers in total) (Elsevier (12%), MDPI (8%), and ACM (8%)). The dominant presence of Google Scholar highlights the role of accessible and inclusive indexing in broadening research reach.

**Figure 9:**
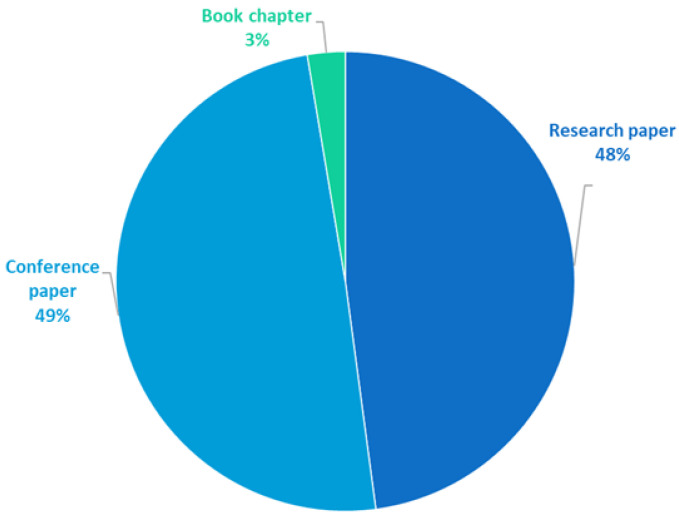
Types of publications.

Regarding the chosen types of paper, the contributions of conference proceedings and research articles (submitted to journals) were almost equal (49% vs. 48%, respectively), with a small number of book chapters (3%), as shown in Figure 9. This clearly shows that much research is still in progress, as the authors want to present their emerging results during conferences (every second paper using Uppaal is a conference proceeding). The strong presence of conference papers also reflects the active and fast-evolving nature of formal verification and real-time systems research, where novel approaches and tool extensions are continuously proposed and evaluated. The results of more advanced (or finished), mature, and comprehensive research are usually published as journal articles (likewise, every second paper). Only a small number of works are book chapters.

### 4.5. RQ5: What Does the Distribution of Research Papers Regarding Geographical Location Look Like?

**Figure 10:**
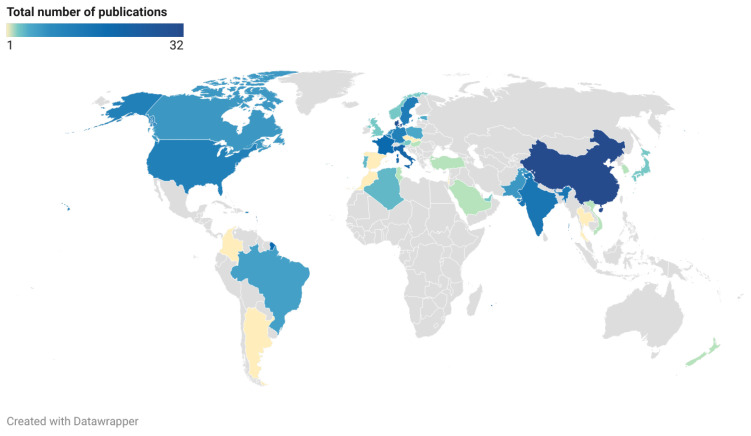
Geographical distribution of papers.

The distribution of publications in terms of the main research countries is summarized in the map in Figure 10. The geographical distribution of the included works reveals a widespread and globally dispersed interest in the application of the Uppaal tool. In particular, the regions in which papers are most frequently published are Southeast Asia, Europe, the Americas, and North Africa, with a particularly high density of research output in technologically advanced and research-intensive countries. China emerges as a pioneer in this field (with 32 papers), which reflects both its growing investment in formal methods and strong academic infrastructure. Denmark, with 30 papers, continues to play a pivotal role, likely due to its foundational contribution to the development and maintenance of Uppaal itself. The other leading countries in this field are Italy (21 papers), France (19 papers), India (15 papers), Sweden (13 papers), Germany (12 papers), the United States of America (11 papers) and Belgium (10 papers). The contributions of the remaining countries are fewer than 10 papers over the considered two-year period. The presence of contributions from regions such as North Africa, South America, and the Middle East highlights the emerging interest and adoption of formal verification tools in developing academic ecosystems. Furthermore, the uneven distribution of publications across countries may also reflect differences in research funding, educational focus, and strategic technological priorities.

## 5. Discussion

Most of the papers using Uppaal take advantage of its capabilities. In justifying its usage, the main aspects mentioned are as follows:

1. Graphical user interface (e.g., [50,58,74,92,97,105,112,131,150,157,205]);
2. Simplicity in model creation (e.g., [50,125]);
3. Powerful simulator and debugger (e.g., [50,97,171]);
4. A powerful verification engine to deliver an absolute guarantee of safety (e.g., [52,106,125,157,198]);
5. Automatic and thorough verification (e.g., [74]).

These match the initial design criteria for the Uppaal tool, that is, its efficiency and ease of use. The authors of [105] even noticed that the interface can also help medical professionals who are not familiar with the software to visualize the overall sys, which indicate that it is user-friendly.Timing aspects are important in many approaches, for example, [58], although there also exist ones that do not utilize the notion of time, for example, [50]. An interesting work [169] in the railway domain summarized many advantages of Uppaal. The authors argue that it can be exploited in the requirement compliance phase for the identification and consolidation of both qualitative and quantitative requirements. The authors of [183] describe Uppaal as the most successful model checker.

### 5.1. A Brief Comparison with Other Mainstream Formal Validation Tools

The conducted systematic literature review reveals that while Uppaal excels in real-time and probabilistic verification, formal validation tools like NuSMV [5] and SPIN [7] have strengths in symbolic and software verification, respectively. PRISM [8] provides robust support for probabilistic systems; nuXmv [6] offers an updated platform for symbolic model checking; and tools like HyTech [10], Ymer [11], and Zing [12] are specialized for hybrid and stochastic systems. In addition to the aforementioned general-purpose model checking tools, there are also specialized tools tailored to specific application domains. For instance, AltaRica [211] is a domain-specific modeling language and toolset developed for safety-critical systems, particularly in the aerospace and industrial sectors. While AltaRica is not a general-purpose model checker, its emphasis on safety and reliability analysis—especially in terms of hazard modeling and fault propagation—makes it highly valuable for model-based safety assessment.

The choice of the tool depends on the specific requirements of the system being analyzed, such as the need for real-time constraints, probabilistic behaviors, or software concurrency. Uppaal is particularly well suited for the modeling and verification of systems in which timing constraints are critical. Its underlying timed automata formalism, coupled with an intuitive graphical interface and efficient verification engine, makes it especially effective for analyzing temporal behaviors under strict time restrictions. Consequently, Uppaal often demonstrates superior performance and usability compared to general-purpose model checkers when applied to real-time or time-sensitive domains.

### 5.2. Exploring the Applicability of *Uppaal* Versions

#### 5.2.1. Classic Uppaal with Symbolic Model Checking

Capabilities and applicability: The basic Uppaal tool environment is, first and foremost, a model checking tool. Its main components are the editor, the simulator, and the verifier. The editor allows users to define input models based on timed automata. These automata are enhanced with additional data types, such as integers and arrays. The models are systems of communicating timed automata, which, in th general cases, are non-deterministic. For such a model, a transition system can be generated. The simulator enables the execution of the given model and it can generate execution paths leading to undesired states. The verifier performs symbolic model checking. Unlike the simulator, it explores the entire state spaces and can produce diagnostic traces when necessary. Different kinds of concurrent real-time systems specify the area of typical applicability of this tool.

Demonstrative case study: In [74] a multi-core processor with a shared cache system is analyzed. The challenge is in verifying the cache consistency protocol to ensure data consistency—a non-trivial task in such parallel systems. However, this system (considered at the RTL level) can be conveniently modeled by communicating finite-state machines (automata). To make the model more suitable for analysis, time aspects are included (timed automata are used). This type of model fits well with the capabilities of classic Uppaal. In the cited work, Uppaal is used for verification of the protocol by means of model checking. This can be considered a typical case study using basic Uppaal.

#### 5.2.2. Uppaal SMC

Capabilities and applicability: Uppaal SMC, as mentioned before, allows us to perform statistical model checking. It provides a very important practical advantage compared to the classical (symbolic) model checking approach. Uppaal SMC randomly simulates a series of executions of its models, then performs a statistical analysis of the obtained behaviors. As long as this approach does not generate complete state spaces, it can be applied for systems which have too complex behavior to be handled as a whole. In addition, it is often reasonable (and much easier) to obtain statistical confirmation of the reliability of the system instead of complete formal proof. SMC can obtain, at its input, stochastic time automaton models, which makes it appropriate to model and analyze failures and other extreme or dangerous situations.

Demonstrative case study: In [97], a framework using Uppaal SMC is presented, in which failure behaviors and attacks are analyzed, allowing for, among others, reliability and cost analyses (including the cost of system repair). A stochastic model called an AFMT (Attack–Fault Maintenance Tree) is developed for this purpose. As a case study, an oil pipeline is considered, where failures such as leakages are analyzed. It can be considered to be a typical application of Uppaal SMC, together with cyber–physical systems or communication systems, which may be the objects of different kinds of attacks and should also be analyzed to determine the reachability of certain states, etc.

#### 5.2.3. Uppaal Stratego

Capabilities and applicability: Uppaal Stratego allows us to develop control strategies with optimized parameters, such as speed, cost, safety, etc., on which a price function depends. In this version of Uppaal, the models assume the existence of a controller that executes a strategy. Here, as in Uppaal SMC, stochastic timed models are used, to which statistical model checking can be applied. However, rather than focusing solely on verification, Stratego facilitates the synthesis of optimal strategies through guided simulation. Different learning methods are available, including reinforcement learning approaches like Q-learning and Monte Carlo Tree Search, which are used to synthesize and refine strategies. An obtained strategy (or a set of strategies) may be either deterministic—providing a unique action for each system state—or non-deterministic—offering multiple viable actions from a given state depending on the optimization criteria or trade-offs.

Demonstrative case study: A representative use of Uppaal Stratego for the control of a heating system based on heat pumps for a family house is presented in [158]. In this scenario, simple simulation and verification of the model were insufficient. Instead, a complex control strategy was required that takes into account the needs of the inhabitants, weather, changing electricity prices, and other parameters. Applying Uppaal Stratego together with a model called EMDP (based on Markov decision processes) allowed the authors to synthesize an efficient controller, which provides energy savings generally better than those provided by the controllers created by alternative methods. Typical applications of Uppaal Stratego include the control of complex hybrid systems that exhibit stochastic behavior and operate under dynamic conditions. These applications often require multi-parameter optimization and have been demonstrated in domains such as autonomous robotics, intelligent transportation systems, and adaptive energy management. The synthesis of control strategies represents a significant capability that extends beyond the scope of classical model checking, which is typically limited to verification rather than strategy generation.

#### 5.2.4. Uppaal TIGA

Capabilities and applicability: The TIGA version shares certain features with Uppaal Stratego: both use models consisting of a controller and a controlled system, and both aim to synthesize strategies. The system behavior is represented as a transition system with controlled transitions (determined by the controller) and uncontrolled ones. This situation can be seen as a game between two players. Timed automatons are used as the primary modeling elements, and a system is modeled as a composition of such automata. One key difference compared to Stratego is that in TIGA, the automatons are not stochastic, and the resulting strategies are deterministic. Uppaal TIGA uses a highly efficient symbolic algorithm to solve timed games, typically not requiring full state-space exploration. Solving a game in this context generally means obtaining a winning strategy (or avoiding a losing one), if such a strategy exists. In the games used in TIGA, unlike in the case of Uppaal Stratego, the aim is not to maximize a continuous cost function, but rather, winning means reaching certain desired states and losing means reaching undesired ones.

Demonstrative case study: In [132,153] a method for synthesizing safe controllers for continuous systems using Uppaal TIGA is described. Integer-valued bounds for the system variables are derived, and states where the variables are beyond such bounds are considered undesired. Uppaal TIGA is then used to synthesize a strategy that avoids these states in the corresponding game model. An industrial case study featured in the paper focuses on a stormwater detention pond. This system, like other bounded continuous systems (such as traffic systems), represents a typical application domain for TIGA.

#### 5.2.5. Uppaal CORA

Capabilities and applicability: Uppaal CORA (CORA is the abbreviation for cost-optimal reachability analysis) is a tool intended to find, in the state space of a given model, one or more paths to the states that satisfy specified conditions. The paths are optimized according to a cost function. The tool is able to find an optimal path (given sufficient time and memory, since this may require extensive state-space exploration) or to obtain a sub-optimal solution by exploring only part of the state space. Uppaal CORA builds on the framework of timed automata used in basic Uppaal, extending it with cost annotations. Is also supports user-defined types and procedures, enhancing modeling flexibility. Uppaal CORA is particularly well suited for solving scheduling and routing tasks, where cost optimization plays a central role.

Demonstrative case study: The Uppaal website (https://uppaal.org/, accessed on 17 May 2025) presents several case studies involving Uppaal CORA, including the vehicle routing problem with time windows (a generalization of the traveling salesman problem with multiple salesmen), the aircraft landing problem, and the energy-optimal task graph scheduling problem. These well-known optimization problems can be effectively addressed by CORA, provided the problem instances are of manageable size. In [190], additional applications are reported, such as job-shop scheduling problems and the power optimization of dataflow applications. Other relevant case studies include programmable logic controllers and smart grid systems. These examples highlight the practical applicability of CORA in domains requiring cost-aware decision-making.

#### 5.2.6. Guidelines for Selecting the Appropriate Uppaal Version

The diverse capabilities of Uppaal make it adaptable to a broad range of application domains. However, each version of Uppaal—be it the classic model checker, the statistical extension (SMC), or the game-based variant (TIGA)—is tailored to address specific modeling needs and verification goals. To support practitioners in selecting the most appropriate version for their context, Table 2 presents a set of general guidelines that summarize the strengths and typical use scenarios of each variant. These guidelines are derived from the analysis of demonstrative case studies and highlight the core features and application conditions that influence tool selection.

### 5.3. Open Challenges

Some research projects indicate drawbacks and open challenges; we have done our best to summarize them from the considered literature:

1. The authors of [28] point out a disadvantage of Uppaal SMC whereby it does not support a hierarchy of states. It is therefore necessary to construct separate templates for the parent-and-child hierarchy in the models used. Despite this fact, the authors still evaluate Uppaal SMC as a promising tool in estimating the probability of satisfying a user-specified performance query and requires much less checking time than traditional formal verification methods.
2. The authors of [57] report that Uppaal Stratego solves limited types of objectives, leading it to make too strong assumptions about the problem.
3. The authors of [115] point out that Uppaal SMC uses the Euler method for solving differential equations, known to be less accurate and entail larger performance overhead in comparison to analytical methods. Moreover, they note that the tool is not optimized for long-lasting simulations.
4. The authors of [131] claim that Uppaal TIGA (1) cannot process parametric timed automata; (2) has no support for shared memory; and (3) requires each model to be consistent.
5. The authors of [132] point out the following regarding Uppaal TIGA: (1) it can only calculate the infimum using symbolic methods; (2) its memory usage seems to be the limiting factor in applying the method to large-scale systems.
6. The authors of [149] faced a problem with the machine power needed to validate the given requirements. Indeed, the verification was not completed due to the state-space problem (it crashed after 20 min on one machine, and after 4 h on the other).
7. The authors of [150] indicate the following regardingUppaal: (1) it could provide a better user experience (according to chatbot developers); (2) its state machine nature limits the size of flows that can be modeled.
8. The authors of [169] provide a wider discussion of Uppaal application in the railway domain. They highlight that due to the standardization of the railway process, it is challenging to determine "how to integrate tools and practices […] and how to adapt the overall workflow to accommodate innovation". Moreover, if Uppaal is meant to be introduced in current industrial processes as T2 tool (the T2 category is dedicated to tools where a fault could lead to an error in verification results), evidence should be provided by the vendors that the results produced by the tool are actually reliable, and that the tool has followed a documented process of development and maintenance. To the knowledge of the authors, this is currently lacking for Uppaal, and this could seriously hamper its adoption.
9. The authors of [170] point out that railway engineers experienced some difficulties in evaluating the results; when Uppaal provided a counterexample, “it proved almost impossible […] to decipher where the error causing the requirement violation was”. The following solution to this problem is suggested: developing a backward mapping/annotating method to show the counterexample in the high-level model.
10. The authors of [180] noted that system variables cannot change via external interactions with the environment, although some other model checkers enable it, but in these cases, the environment must also be modeled.
11. The authors of [192] mention that the query language for requirement specification in Uppaal is less expressive than that of Timed Computation Tree Logic (TCTL), and thus, not every TCTL formula can be expressed in Uppaal. Moreover, they indicate some problems with (1) timed temporal operators; (2) the nesting of model operators; and (3) unavailability of the weak-until operator.
12. The authors of [198] indicate that “the public, academic version […] is unable to exploit the computing potential of current shared-memory multi-core machines”.
13. The authors of [207] state that a limitation in the area of clock synchronization algorithm verification is that Uppaal does not permit the reading of values of the clock variables.

Despite its strengths, several open challenges are indicated by researchers in the usage of Uppaal. One of the mentioned limitations is the lack of support for state hierarchies in Uppaal SMC, requiring separate templates for parent and child states. It also faces some issues with the Euler method for solving differential equations, leading to increased performance overhead and reduced accuracy, particularly in long-duration simulations. Uppaal Stratego is restricted to a narrow set of objectives, often requiring strong assumptions. Uppaal SMC and TIGA have limitations such as the inability to process parametric timed automata, a lack of support for shared memory, and scalability challenges related to memory usage. Other concerns include state-space explosion causing verification failures, the inability to interact with system variables externally, and less expressive query languages compared to alternatives like TCTL. Furthermore, Uppaal struggles to fully utilize modern multi-core processors and lacks the ability to verify clock synchronization algorithms effectively. These challenges highlight the need for further improvements, particularly in scalability, user experience, and domain-specific applications (such as railway systems), which could significantly broaden its utility in real-world, large-scale, and complex systems.

### 5.4. Possible Solutions

While Uppaal has proven to be a powerful tool for model checking and the verification of real-time and safety-critical systems, several challenges remain that hinder its broader applicability and efficiency. However, there are several promising solutions that could address these limitations and enhance the tool’s performance and usability.

One significant challenge is the lack of support for hierarchical states in Uppaal SMC. This limitation forces users to create separate templates for parent and child states, complicating model construction. A potential solution would be to extend the modeling framework to incorporate native support for hierarchical state machines, streamlining the modeling process. Similarly to the case with UML state machine diagrams, the use of a hierarchy would offer many new possibilities and more flexibility to the designer. Now, if the specification is written in a hierarchical form, it has to first be “flattened” before it can be processed further [212]. Another open issue is that the use of the Euler method for solving differential equations in Uppaal SMC is known to introduce significant performance overhead and accuracy issues, particularly in long-duration simulations. One potential improvement could be to integrate more accurate numerical solvers and optimize the tool for parallel and distributed computing. These advancements could improve both the accuracy and efficiency of simulations, especially in complex systems.

Another challenge lies in the limited set of objectives supported by Uppaal Stratego, which often requires strong assumptions about the system being modeled. To broaden its applicability, future versions of Stratego could integrate more flexible optimization techniques or adopt a general-purpose planning framework, allowing it to handle a wider range of objectives. In the case of Uppaal TIGA, several limitations affect its ability to handle large-scale systems, such as the inability to process parametric timed automata and a lack of support for shared memory. Enhancing this tool version to address these limitations, together with improvements in memory optimization and symbolic methods, could significantly improve its scalability and versatility in complex systems.

State-space explosion and excessive resource demands have been reported as significant challenges when verifying large systems in Uppaal. Addressing this issue could involve the incorporation of advanced state-space reduction techniques, such as abstraction or symbolic state-space exploration. Additionally, parallelization strategies and better exploitation of multi-core and distributed computing architectures would help improve performance and reduce computational overhead. In terms of user experience, the modeling approach imposes limitations on the size and complexity of the models that can be handled. Improving the graphical user interface and supporting more flexible modeling paradigms would make the tool more accessible and suitable for large-scale models, thus enhancing the user experience. Another usability issue arises when Uppaal generates counterexamples that are difficult to interpret, making it challenging for engineers to identify the cause of requirement violations. Developing a backward mapping or annotation feature would allow users to trace counterexamples back to the high-level model, providing greater insight into the verification results.

Uppaal also currently lacks the ability to interact with system variables externally, limiting its flexibility compared to other model checkers. Extending the tool to allow for external system interaction would make it more suitable for modeling systems that interact with real-world environments, thus increasing its applicability to a wider range of scenarios. Regarding the expressiveness of the query language, it is currently less expressive than Timed Computation Tree Logic (TCTL), which may restrict the types of properties that can be modeled and verified. Enhancing the query language of Uppaal to support a wider range of temporal operators would provide users with greater flexibility in specifying system requirements. Finally, Uppaal’s inability to fully utilize modern multi-core processors and its reliance on less efficient numerical methods for clock synchronization verification remain significant limitations. Optimizing the underlying algorithms to better exploit multi-core and shared-memory architectures, as well as improving clock synchronization handling, would help address these issues and improve its performance in large, time-sensitive systems.

For domain-specific applications, particularly in the railway sector, Uppaal has faced integration challenges due to standardization issues and the lack of documented validation processes. Future versions could work on providing a robust framework for integrating Uppaal with industry standards, with the development of clear documentation regarding the tool’s reliability and validation processes. This would help address concerns about its adoption in industrial settings.

These proposed solutions not only address the specific challenges identified in the literature, but also provide a path forward for enhancing the capabilities of the Uppaal tool in various domains. By implementing these improvements, Uppaal could further solidify its position as a leading tool for formal verification in real-time and safety-critical systems.

## 6. Conclusions

This study presents a systematic review of the literature on the application areas of the Uppaal tool. It has been shown that its comprehensive features make it suitable for potential use in various fields. The study included 188 articles published in 2022 and 2023. The results clearly indicate that the most popular version is Uppaal SMC, which supports statistical model checking, followed by Uppaal Stratego, dedicated to strategy analysis. The distribution of works between conference papers and research papers is almost equal, which suggests that many research projects are still ongoing (the preliminary results are usually first presented at conferences). This is very promising for the near future. The most frequently publishing regions are Southeast Asia, Europe, and the Americas.

Five research questions were defined in this study, corresponding to (1) the application areas of the Uppaal tool, (2) the popularity of its versions, (3) the most popular keywords in obtained papers, (4) the distribution of articles regarding access options, scientific databases, and publication types, and (5) the distribution of papers in terms of geographical location. All of these questions have been thoroughly answered in this paper. This allows us to provide summaries and insights into possible further developments of the Uppaal tool. The literature analysis shows that the choice of the Uppaal tool often results from its ease of use and high efficiency. These aspects are often emphasized in various application areas, since Uppaal is frequently used by non-engineers. This aligns with the original design goals of Uppaal, prioritizing user accessibility and computational performance. For instance, studies have highlighted the intuitive graphical interface as beneficial not only to software engineers but also to medical professionals, enabling broader interdisciplinary collaboration. It follows that these aspects should still be the key design issues for developers. Some of the research papers point out the imperfections of the tool, such as limited support for hierarchical modeling, restricted objective types in strategy synthesis, higher performance overhead due to numerical methods, and challenges with scalability and usability. This information may be of great importance to programmers and engineers involved in software development.

The main limitation of this study is that only publications written in English were taken into account. Some preliminary results published in other languages, for example, those presented at local conferences, may therefore have been omitted.

## Figures and Tables

**Table 1:** Most notable sectors of Uppaal usage.

Domain	Research Papers
Autonomous systems	[23,24,25,26,27,28]
Blockchain	[29,30,31,32,33]
Communication networks	[34,35,36,37,38,39,40,41,42,43,44]
Cyber–physical systems	[45,46,47,48,49,50,51,52,53,54,55,56]
Cybersecurity	[57,58,59,60,61,62,63,64,65,66,67,68,69,70,71,72,73]
Electronics	[74,75,76]
Embedded systems	[77,78,79,80,81,82,83,84,85,86,87]
Industry	[88,89,90,91,92,93,94,95,96,97,98,99,100,101]
Machine learning	[102,103,104]
Medicine	[105,106,107,108,109,110,111,112,113,114,115,116]
Power systems	[117,118,119,120,121,122,123,124,125]
Real-time systems	[126,127,128,129,130,131,132,133]
Robotics	[134,135,136,137,138,139,140,141,142,143]
Software	[144,145,146,147,148,149,150,151,152,153,154,155,156,157]
Thermal dynamics	[158,159,160,161]
Train and railway engineering	[162,163,164,165,166,167,168,169,170,171,172,173,174,175,176,177]
User journeys	[178,179]
Verification	[180,181,182,183,184,185,186,187,188,189,190,191,192,193,194,195,196,197]

**Table 2:** Guidelines for selecting the appropriate Uppaal variant.

Use Case/Domain	Recommended Version	Key Features Needed	Notes
Formal verification of real-time systems	Classic	Timed automata, reachability analysis, exhaustive model checking, safety and liveness properties	Ideal for protocol verification, embedded systems, and communication systems
Systems with stochastic behavior or uncertainty	SMC	Statistical model checking, probability evaluation, simulation	Suitable for energy-aware systems, battery analysis, and performance evaluation under uncertainty
Adaptive control in smart systems; resource-aware decision-making; energy-aware scheduling	Stratego	Strategy synthesis, cost optimization, machine learning integration	Suitable for systems requiring optimal and adaptable strategies; leverages reinforcement learning to improve control performance
Adversarial control; planning under uncertainty	TIGA	Timed game automata, strategy synthesis, controller generation	Useful in scheduling, autonomous systems, and human–robot interaction
Real-time scheduling; performance evaluation of timed systems; cost-optimal planning	CORA	Cost variables, optimal scheduling, extended priced timed automata	Ideal for scenarios where timing and resource consumption must be optimized simultaneously
